# Evaluating the effectiveness of different topical anesthetic methods in reducing pain during intravitreal injections: a systematic review and meta-analysis

**DOI:** 10.1186/s40942-026-00892-5

**Published:** 2026-06-30

**Authors:** Renad Alhomidi Almotiri, Zahraa Hussain Alsaggaf, Yara Hamed Abosabaah, Sadeem Saad Abdulaziz Alkaluf, Rana Ali A. Algahamdi, Ali Mousa Ahmed Alkhalaf, Ayah Mazen Kurdi, Ishraq Tail Alkhudaidi, Sarah Husam AlJefri

**Affiliations:** 1 College of Medicine, Imam Mohammad Ibn Saud Islamic University (IMSIU), Riyadh, Saudi Arabia; 2 Department of Ophthalmology, Jeddah Eye Hospital, Jeddah, Saudi Arabia; 3 College of Medicine, King Saud bin Abdulaziz University for Health Science, Riyadh, Saudi Arabia; 4 College of Medicine, King Faisal University, Al- Ahsa, Saudi Arabia; 5 College of Medicine, Al-Baha University, Al- Baha, Saudi Arabia; 6 College of Medicine, King Abdulaziz University, Jeddah, Saudi Arabia; 7 Makkah Health Cluster, Makkah, Saudi Arabia; 8 Department of Ophthalmology, College of Medicine, Imam Mohammad Ibn Saud Islamic University (IMSIU), Riyadh, Saudi Arabia

**Keywords:** Local anesthesia, Topical anesthesia, Pain control, Pain management, Intravitreal injections, Anti-vascular endothelial growth factor

## Abstract

**Background:**

The rapid rise in intravitreal injections (IVIs) for vitreoretinal disorders underscores the need to optimize the procedure and support long-term patient compliance. Patient-reported pain and discomfort remain significant barriers to adherence. This systematic review evaluates the efficacy and safety of various local anesthetic methods for mitigating pain during IVIs.

**Methods:**

Conducted in accordance with PRISMA guidelines, a comprehensive search of PubMed and Google Scholar was performed to identify English-language randomized controlled trials (RCTs). Eligible studies included patients aged 18 years and older receiving local anesthesia for IVIs, where pain was quantified using standard visual or oral analog scales (0–10 or 0–100).

**Results:**

Our systematic review and meta-analysis included 14 RCTs comprising 1,300 patients. Analysis of topical lidocaine, tetracaine, and proparacaine formulations showed comparable analgesic efficacy, with all agents consistently achieving mild mean Visual Analog Scale (VAS) pain scores. No clinically meaningful superiority was identified among these primary agents (*p* = 0.62). Adjunctive therapies, particularly physical cooling (e.g., ice patches), demonstrated significant pain-reducing potential. While oxybuprocaine alone was less effective, its combination with physical cooling achieved the lowest mean pain scores. Meanwhile, topical NSAIDs showed limited additional benefit. Regarding safety, subconjunctival anesthetic injections were associated with a significantly higher incidence of subconjunctival hemorrhage and chemosis compared to topical applications, suggesting that topical strategies provide a favorable safety and efficacy profile for routine intravitreal injections.

**Conclusion:**

In conclusion, this systematic review and meta-analysis confirms that no single topical anesthetic is superior for intravitreal injections; all primary agents provide comparable, effective pain control. Topical application is safer than subconjunctival injection, which is associated with higher rates of hemorrhage and chemosis. Consequently, clinical protocols should standardize low-risk topical methods and incorporate simple non-pharmacological adjuncts, such as physical cooling, to optimize patient comfort.

## Introduction

Intravitreal injections (IVIs) are considered one of the most significant advances in the management of vitreoretinal disorders and have become the standard of care for delivering therapeutics to the retina [1]. The number of IVIs delivered has increased exponentially with the introduction of anti-vascular endothelial growth factors (anti-VEGF) [1, 2]. In the USA alone, it’s estimated that over 7 million IVIs are administered annually, and this number is expected to rise [3]. In addition, a study conducted in Norway revealed a 100-fold increase in the annual anti-VEGF given from 2006 to 2018 [4]. Furthermore, Anti-VEGF is utilized in many retinal diseases such as age-related macular degeneration, diabetic retinopathy, and retinal occlusion [1]. The treatment of most of these pathologies frequently requires multiple IVIs over an indeterminate course of time [5].

Given the increasing demand and the long-term nature of treatment, optimizing the patient experience during the procedure is essential, and to ensure the patient’s adherence to the treatment. One important aspect is the pain and discomfort the patient may experience during IVI, which in turn can be alleviated using local anesthesia [6, 7]. There are various anesthetic agents and delivery methods that can be used [6]. However, to the best of our knowledge, there is no consensus regarding the best anesthetic substance and technique [8].

Therefore, the aim of this review is to evaluate the efficacy of different anesthetic methods for intravitreal injections.

## Methods & materials

### Literature search

In our systematic review and meta-analysis, we aimed to evaluate the effectiveness of various topical anesthetic methods in diminishing the pain associated with intravitreal injections. This review was registered with PROSPERO (ID: CRD42024516713) and adhered strictly to its prescribed methodology. The search strategy was done using keywords as follows, ‘Topical Anesthetic,’ ‘Local anesthesia,’ ‘Topical anesthesia,’ ‘anesthesia,’ ‘Pain,’ ‘Pain control,’ ‘Pain management,’ ‘Intravitreal Injections,’ ‘IVI,’ ‘Anti-vascular endothelial growth factor,’ ‘anti-VEGF,’ and ‘Steroids in IVI.’ These terms were used individually and in combination. A systematic search of electronic databases including Google Scholar and PubMed was conducted in April 2026.

### Study selection

We included English-language studies, which studied patients aged 18 and above who had received topical anesthesia during intravitreal injections. No initial publication date restrictions were applied during our search. The scope was limited to randomized controlled trials that compared the effect of different local anesthetic techniques for intravitreal injections, with pain quantified using analog (visual or oral) scales ranging from 0 to 10 or 0 to 100. We excluded non-English studies, pediatric studies (ages 0–18 years), non-randomized clinical trials, and publications classified as reviews, letters, conference abstracts, or editorials. Studies that involved sedation or general anesthesia during intravitreal injections, assessed interventions other than anti-VEGF agents or corticosteroids, or used pain scales outside the predefined range were also excluded.

The screening process was managed using Rayyan (Rayyan (https://www.rayyan.ai/)). While Rayyan includes AI-assisted features, these tools were not utilized in this study. Full-text articles were screened manually and independently by (A.A, A.K, S.A, and Y.A) to determine eligibility. Any disagreements were resolved through discussion, with a fifth reviewer (R.A) consulted when necessary.

### Data extraction

The data from each report were retrieved by 5 investigators (R.A, A.A, A.K, S.A, and Y.A). Variables collected include study attributes such as authorship, year of publication, study country, design, cohort size, and follow-up duration. Information regarding the type of anesthetic as well as the studies’ inclusion and exclusion criteria were included. Patient demographics including age, sex, medical antecedents, and concurrent medications were also extracted. Intervention details involving anesthetic dosage, the time of administration, and application methods, alongside outcome measures that assessed the comparative efficacy of different anesthetics and any adverse effects documented were added. Furthermore, statistical methods used, effect size, confidence intervals, heterogeneity, and publication bias were assessed. Each study’s primary conclusion, limitations, and recommendations were also extracted.

Quality assessment was based on the revised Cochrane risk-of-bias tool for randomized trials. A pair of reviewers (I.A and Z.A) independently evaluated the risk of bias for each article. Disagreements between reviewers were reconciled through discussion and consultation with a third expert reviewer when necessary.

### Statistical analysis

The efficacy of different anesthetic agents and administration strategies was evaluated using analog pain scales, including visual or oral analog scales ranging from 0 to 10 or 0 to 100. When necessary, pain scores reported on a 0-100 scale were converted to a 0–10 scale to ensure consistency across studies.

Pain scores were extracted as continuous variables using the mean, standard deviation (SD), and sample size for each eligible treatment arm, and were pooled as raw mean VAS scores (MRAW) on the 0–10 scale.

Because the included studies were predominantly single-arm and varied in anesthetic agent, concentration, formulation, timing, and administration protocol, random-effects models were applied throughout. For continuous pain outcomes, pooled raw means were estimated using the restricted maximum likelihood (REML) estimator for between-study variance. Hartung-Knapp adjustment was applied for the calculation of random-effects confidence intervals. Heterogeneity was assessed using the I² statistic, τ², and Cochran’s Q test. Prediction intervals were calculated where appropriate to estimate the expected range of effects in future comparable studies.

For safety outcomes, the incidence of subconjunctival hemorrhage was pooled as a proportion using random-effects meta-analysis. Sensitivity analyses were conducted to evaluate the robustness of the pooled estimates. Leave-one-out analyses were conducted by sequentially omitting each study from the model. Additional outlier-exclusion analyses were performed for treatment arms identified as major contributors to heterogeneity, including the 1% tetracaine arm and the 10% lidocaine gel arm.

All analyses were conducted in R software (version 4.6) using the meta package for single-arm pooling and forest plots. Metafor package was used for prediction intervals where appropriate.

### Risk of bias

Out of the 14 included RCTs, seven articles were classified as low risk of bias, four as high risk, and three as having some concerns. The details of bias assessment in each domain can be seen in (Table 1).

**Table 1:** Risk-of-bias assessment for each included study

References	Bias arising from the randomization process	Bias due to deviations from intended interventions	Bias due to missing outcome data	Bias in measurement of the outcome	Bias in selection of the reported result	Overall risk of bias
Sanabria, Maria R. et al. (2013) [9]	No information\ High	Low	Low	Low	Low	High
Makri, Olga E. et al. (2018) [10]	Low	Low	Low	Low	Low	Low
Örnek, Nurgül et al. (2014) [11]	No information\ likely low	Low	Low	Low	Low	Low
Davis, Michael J. et al. (2012) [12]	No information\ likely low	Low	Low	Low	Low	Low
Georgakopoulos, Constantine D. et al. (2012) [13]	Low	Low	Low	Low	Low	Low
Rifkin, Lana et al. (2012) [14]	No information	Low	Low	Low	Low	Some concerns
Yau, Gary L. et al. (2010) [15]	Low	Low	Low	Low	Low	Low
Kozak, Igor et al. (2005) [16]	High	Low	Low	Low	Low	High
Shiroma, Helio F. et al. (2014) [17]	No information/ likely low	Low	Low	Low	Low	Low
Reynolds, Margret et al. (2020) [18]	No information	Some concerns	Low	Some concerns	Low	High
Wingelaar, Maxwell J. et al. (2021) [19]	Low	Low	Low	Low	Low	Low
Blaha, Gregory R. et al. (2011) [20]	Some concerns	Some concerns	Some concerns	Low	Some concerns	High
Lo, Jeffrey Man Yeung et al. (2025) [21]	Low	Low	Low	Low	Some concerns	Some concerns
Yahalomi, Tal et al. (2026) [22]	Low	Some concerns	Low	Low	Low	Some concerns

## Results

### Primary results of the search

**Fig. 1:**
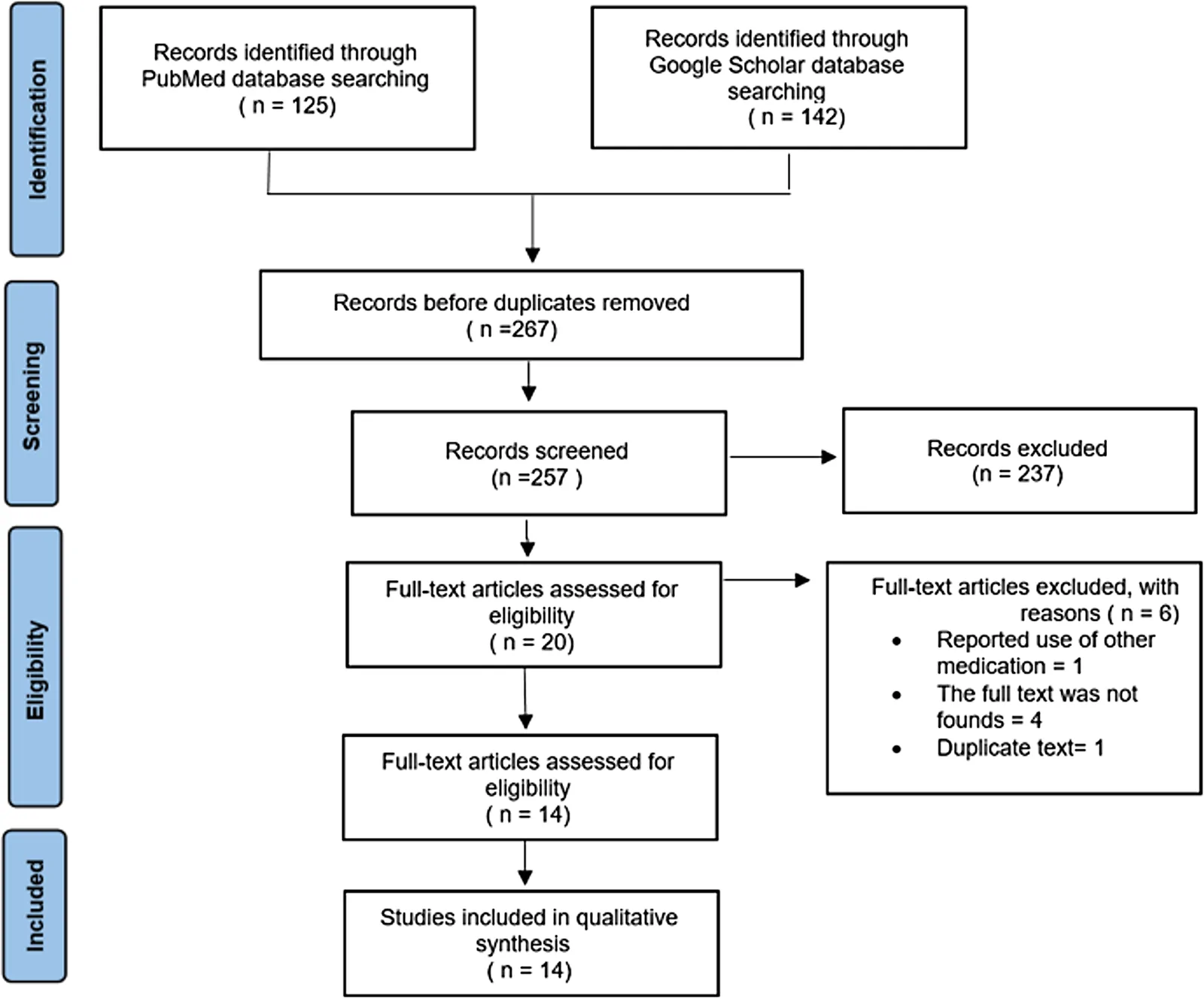
A flowchart showing the selection of studies based on preferred reporting items for systematic reviews and meta-analysis (PRISMA)

Our initial search of two databases, PubMed and Google Scholar, yielded a total of 267 papers. After removing 10 duplicates, we screened the remaining 257 papers by reading titles and abstracts (Fig. 1). Subsequently, we excluded 237 papers and retrieved the full text for 20 papers to assess their eligibility. Ultimately, only 14 articles met all the necessary criteria and were incorporated into our systematic review. These included studies encompassed a range of designs, such as prospective randomized double-blinded placebo-controlled crossover interventional studies, prospective randomized controlled trials, and prospective, randomized, double-masked, placebo-controlled clinical trials. Furthermore, these studies were published between 2005 and 2026 and originated from various countries, which can be seen in (Table 2).

**Table 2:** Characteristics of the studies included

References	Study Design	Country	Sample Size
Sanabria, Maria R. et al. [9]	Prospective, randomized, double-masked, comparative study.	Spain	156
Makri, Olga E. et al. [10]	Prospective, randomized, double-blinded, placebo-controlled, crossover interventional study.	Greece	55
Örnek, Nurgül et al. [11]	Prospective, randomized study.	Turkey	96
Davis, Michael J. et al. [12]	Prospective randomized clinical trial.	United States	120
Georgakopoulos, Constantine D. et al. [13]	Prospective, randomized, double-masked, controlled study.	Greece	30
Rifkin, Lana et al. [14]	Prospective, interventional, randomized study.	United States	60
Yau, Gary L. et al. [15]	Prospective, randomized, triple-armed, double-blinded, prospective, single-centered trial.	Canada	97
Kozak, Igor et al. [16]	Prospective randomized clinical trial.	United States	28
Shiroma, Helio F. et al. [17]	Prospective clinical trial.	Turkey	260
Reynolds, Margaret et al. [18]	Prospective, randomized controlled trial.	United States	36
Wingelaar, Maxwell J. et al. [19]	Prospective, randomized, double-masked, placebo-controlled clinical trial	United States	46
Blaha, Gregory R. et al. [20]	Prospective, randomized clinical trial.	United States	28
Lo, Jeffrey Man Yeung et al. [21]	Prospective, randomized, double-blinded crossover study.	China	63
Yahalomi, Tal et al. [22]	prospective, interventional, double-blinded, randomized controlled trial.	Israel	239

### Patient profiles and characteristics

A total of 1,314 patients were identified, of whom 1,300 were included in the analysis after exclusion of 14 patients. The fourteen patients were excluded due to unsuccessful blinding (*n* = 3) [10], refusal to participate (*n* = 4) [15], dropping out due to financial restraints of IVI (*n* = 2) [21] and from discomfort associated with the first injection (*n* = 1) [21]. Additionally, four patients (*n* = 4) [20] were excluded because the study closed after completion of the 24 randomized block sequences; only patients who completed these sequences were included in the final analysis.

**Table 3:** Intervention and reported outcomes

References	Type of Intervention	Indications for Treatment	Pain score scale and Average pain score	Outcomes
Sanabria, Maria R. et al. [9]	→*Pre-IVI*: - **Group A**: Tetracaine 0.5%+ naphazoline 0.05% drops. **-Group B**: Lidocaine 5% drops. →*Post-IVI*: **-Protocol 1**: Tobramycin 0.3% drops. **-Protocol 2**: Tobramycin 0.3% and diclofenac 0.1% drops.	AMD, Myopic CNV, DME, RVO, PCV, NVI and CME.	Pain was quantified using a numerical rating pain scale ranging from 0 to 10. **Immediately post-IVI**: **Group A**: 2.85 **Group B**: 2.67 **24 h post-IVI**: Protocol 1: 1.75 Protocol 2: 1.75	Topical tetracaine and lidocaine provide similar anesthesia immediately after the IVI (*p* = 0.73), and at 24 h (*p* = 0.979). Topical diclofenac does not seem to reduce pain scores after IVI (*p* = 0.46).
Makri, Olga E. et al. [10]	**-Group A**: Topical nepafenac 0.1%. **-Group B**: Artificial tears as a placebo.	AMD, DME and RVO.	The VAS measures pain on a scale of 0 to 10. **Immediately post-IVI**: **Group A**: 1.58 **Group B**: 3.32 **6 h post-IVI**: **Protocol 1**: 1.5 **Protocol 2**: 3.75	The VAS pain score was statistically significantly lower immediately and 6 h post-IVI in patients treated with nepafenac. (*p* = 0.001 and < 0.001 respectively).
Örnek, Nurgül et al. [11]	**-Group A**: Levobupivacaine 0.75% drops. **-Group B**: Proparacaine 0.5% drops.	CME and AMD	The VAS used a scale of 0 to 100. **Immediately post-IVI**: **Group A**: 44.77 ± 16.42.3 **Group B**: 34.18 ± 14.83.3	Topical proparacaine 0.5% was more effective in preventing pain during intravitreal injections (*p* = 0.003).
Davis, Michael J. et al. [12]	**-Group A**: Proparacaine hydrochloride 0.5% drops **-Group B**: Proparacaine 0.5% drops followed by 4% lidocaine-soaked cotton-tipped swabs. **-Group C**: Lidocaine hydrochloride 3.5% ophthalmic gel drop.	****	Pain scores were graded using a standardized scale of 0 to 10. **Lid Speculum Pain**: **Group A**: 0.85 **Group B**: 0.50 **Group C**: 0.65 **Needle Stick Pain**: **Group A**: 1.78 **Group B**: 1.75 **Group C**: 1.48 **Burning Sensation Pain from the povidone-iodine**: **Group A**: 1.45 **Group B**: 1.58 **Group C**: 1.13	There were no statistically significant differences in pain scores between the three anesthetic groups. Lid Speculum Pain: (*p* = 0.32). Needle Stick Pain: (*p* = 0.38). Burning Sensation Pain from the povidone-iodine: (*p* = 0.23).
Georgakopoulos, Constantine D. et al. [13]	All patients received tetracaine 0.5% drops, and either: **-Group A**: Ketorolac trometamol 0.5% drops. **-Group B**: Placebo (vehicle solution).	AMD	The pain score results were measured immediately using the 0 to 100 VAS. **Immediately post-IVI**: **Group A**: 8.16 ± 1.3 **Group B**: 12.33 ± 1.41	Topical ketorolac 0.5% reduces patients’ pain perception during intravitreal drug administration (*p* = 0.0003)
Rifkin, Lana et al. [14]	**-Group A**: Tetracaine HCl 0.5% gel. **-Group B**: Proparacaine HCl ophthalmic solution. **-Group C**: Tetracaine HCl ophthalmic solution.	DME, AMD and CRVO.	Pain scores were quantified using a VAS score ranging from 0 to 10 **15 min post-IVI**: **Group A**: 3.39 ± 2.26 **Group B**: 3.17 ± 2.18 **Group C**: 3.05 ± 2.01	Patients receiving tetracaine reported a statistically significant lower pain score than patients receiving proparacaine or Tetracaine HCl 0.5% gel (*p* < 0.01).
Yau, Gary et al. [15]	**-Group A**: Tetracaine hydrochloride 0.5% drops with a lidocaine hydrochloride 4% pledget. **-Group B**: Tetracaine hydrochloride 0.5% drops alone. **-Group C**: Cocaine 4% (with epinephrine 1/100,000) drops alone.	AMD	Mean pain scores were calculated from 100-mm VAS measurements taken immediately and 15 min post-IVI. **Group A**: 19 **Group B**: 21 **Group C**: 21	There was no clinical difference in patient pain experience between the three anesthetic options tested (*p* = 0.549)
Kozak, Igor et al. [16]	**-Group A** (Crossover Group where patients received both interventions on different occasions.) **-Group B** (Single intervention Group) **-Intervention 1**: Lidocaine 2% subconjunctival injection. **-Intervention 2**: Lidocaine 2% gel.	AMD	The study used a descriptive and numerical pain analog scale to assess pain from 0 to 10. **Group 1 (Crossover)**: **Intervention 1**: 1.83 ± 0.75 **Intervention 2**: 1.58 ± 0.68 **Group 2 (Single Treatment)**: **Intervention 1**: 1.62 ± 0.39 **Intervention 2**: 1.50 ± 0.39	There was no significant difference between the interventions in Group 1 (*p* = 0.67), and Group 2 (*p* = 0.82). However, patients noted discomfort or pain during the subconjunctival anesthetic injection process, whereas they experienced no pain from the application of the gel.
Shiroma, Helio F. et al. [17]	**-Group A**: Lidocaine 2%. **-Group B**: Lidocaine 3.5%. **-Group C**: Lidocaine 5%. **-Group D**: Lidocaine 8%. **-Group E**: Lidocaine 12%.	AMD, DME and RVO	Pain scores were measured using a VAS from 0 to 10 **15 min post-IVI**: **Group A**: 2.63 (± 1.68) **Group B**: 2.08 (± 1.35) **Group C**: 2.00 (± 1.65) **Group D**: 1.93 (± 1.40) **Group E**: 1.83 (± 1.35)	Statistical analysis indicated that the anesthetic effect was similar across all groups, and there was no significant difference in pain scores despite the varying concentrations (*p* = 0.077).
Reynold, Margaret et al. [18]	**-Group A**: Proparacaine 0.5% with preservative benzalkonium chloride 0.01%. **-Group B (Control)**: povidone iodine 5% (PI).	AMD	Pain scores were measured by numerical rating scale ranging from 0 to 10. **At the moment of injection**: **Group A**: 1 **Group B**: 2 **Pain remaining shortly after the procedure**: **Group A**: 0 **Group B**: 1	Patient experience did not significantly differ between groups. **At the moment of injection**:(*p* = 0.25). **Pain remaining shortly after the procedure**:(*p* = 0.86).
Wingelaar, Maxwell. J et al. [19]	**-Group A**: Bromfenac 0.09% before the injection and one propylene glycol 0.6% (placebo) after the injection. **-Group B**: Propylene glycol 0.6% (placebo) before the injection and one drop of bromfenac 0.09% after the injection. **-Group C (Control)**: Propylene glycol 0.6% (placebo) both before and after the injection.	****	The mean pain scores using pain scale (0 to 10) reported for each group were: **Group A**: **Immediately after IVI**: 0.75 **At 6 h**: 0.53 **At 24 h**: 0.00 **Group B**: **Immediately after**: 1.13 **At 6 h**: 0.07 **At 24 h**: 0.00 **Group C**: **Immediately after**: 0.53 **At 6 h**: 0.13 **At 24 h**: 0.00	The standard anesthetic combination provided effective pain control for up to 24 h post-injection. The addition of bromfenac 0.09% did not result in any clinically significant difference in pain reduction.
Blaha, Gregory R. et al. [20]	**-Group A**: Proparacaine 0.5% drops **-Group B**: Tetracaine 0.5% drops. **-Group C**: Lidocaine 4% pledget. **-Group D**: Subconjunctival injection of lidocaine 2%.	AMD	Pain scores measured immediately via a 10-point analog pain rating scale: **Average Injection Pain Scores**: **Group A**: 2.8 **Group B**: 3.1 **Group C**: 3 **Group D**: 2.3 **Average Anesthesia Pain Scores**: **Group A**: 0.7 **Group B**: 1 **Group C**: 1.4 **Group D**: 1.6 **Combined Pain Scores (Anesthesia + Injection)**: **Group A**: 3.5 **Group B**: 4.1 **Group C**: 4.4 **Group D**: 3.8	There was no significant difference (*p* = 0.28) between these methods.
Lo, Jeffrey Man Yeung et al. [21]	All patients in both groups first received standard topical proparacaine 0.5% drops. **-Group A**: Proparacaine 0.5% pledget. **-Group B**: Placebo Group (normal saline pledget).	AMD, DME, CRVO, BRVO, Macular telangiectasia.	Pain scores were measured using a VAS (0–10). **Immediately after IVI**: **Group A**: 2.80 ± 2.54. **Group B**: 2.51 ± 2.08 **10 min after IVI**: **Group A**: 2.13 ± 2.51 **Group B**:1.81 ± 2.00 **20 min after IVI**: **Group A**: 1.65 ± 2.34 **Group B**:1.23 ± 1.51	The study overall did not show any statistically significant additional analgesic benefit when adding a pledget to topical anesthetic drops. **Immediately after IVI**: (*p* = 0.48). **10 min after IVI**: (*p* = 0.43). **20 min after IVI**: (*p* = 0.24).
Yahalomi, Tal et al. [22]	**-Group A**: Lidocaine gel 3%. **-Group B**: Lidocaine gel 10%. **-Group C**: Oxybuprocaine 0.4% drops. **-Group D**: Tetracaine HCl 1% drops. **-Group E**: Combined oxybuprocaine 0.4% eye drops and an ice patch applied to the closed eyelid for two minutes.	AMD, DME, PDR, RVO and CME	The study evaluated pain using a VAS ranging from 0–10. **1 min post-IVI**: **Group A**: 7.6 **Group B**: 7.5 **Group C**: 6.66 **Group D**: 2.16 **Group E**: 1.42 **10 min post-IVI**: **Group A**: 7.56 **Group B**: 7.42 **Group C**: 5.6 **Group D**: 1.44 **Group E**: 1.06	Pain scores differed significantly between groups at both theone-minute (*p* < 0.001) and ten-minute (*p* < 0.001) marks. Tetracaine and the oxybuprocaine/ice patch combination demonstrated the lowest mean pain scores, while the lidocaine 10% group had the highest mean pain score.

**** indicates missing information in the corresponding paper

*AMD*: age-related macular degeneration, *DME*: diabetic macular edema, *RVO*: retinal vein occlusion, *CRVO*: central retinal venous occlusion, *BRVO*: branched retinal venous occlusion, *CNV*: choroidal neovascularization, *IVI*: intravitreal injection, *VAS*: visual analogue scale, *CSCR*: central serous chorioretinopathy, *PCV*: polypoidal vasculopathy, *CME*: cystoid macular edema

Table 3 summarizes various studies evaluating different anesthetic interventions for intravitreal injections, focusing on pain scores and treatment outcomes. The studies compare multiple local anesthetic agents, including proparacaine, tetracaine, lidocaine (in various formulations), levobupivacaine, bupivacaine, and oxybuprocaine. As well as assessing adjunctive non-steroidal anti-inflammatory drugs (NSAIDs) such as ketorolac, nepafenac, and bromfenac, in their effectiveness in reducing pain associated with the procedure.

### Detailed IVI protocol

Sanabria et al. [9] included 156 patients. Eighty-six patients were randomized to regimen A (0.5% tetracaine with naphazoline) and seventy patients to regimen B (5% lidocaine). Additionally, seventy-eight patients were assigned to each of the post-injection topical protocols.

Makri et al. [10] included 52 patients scheduled to undergo IVIs of anti-VEGF. Patients were randomized in a 1:1 ratio to receive topical nepafenac 0.1% or artificial tears as a placebo one hour before subsequent IVIs.

Ornek et al. [11] enrolled 96 consecutive patients into two groups receiving either topical levobupivacaine 0.75% (*n* = 48) or proparacaine 0.5% (*n* = 48).

Davis et al. [12] included 120 sequential patients undergoing intravitreal injections randomized to one of three groups: proparacaine hydrochloride 0.5% (*n* = 40), proparacaine hydrochloride plus 4% lidocaine-soaked cotton-tipped swabs (*n* = 40), or 3.5% lidocaine ophthalmic gel (*n* = 40).

Constantine D.et al. [13] included 30 patients who received topical ketorolac 0.5% or vehicle prior to intravitreal drug administration. All procedures used tetracaine 0.5% drops as baseline anesthesia before the injection.

Yau et al. [15] included 93 patients randomized 1:1:1 to receive 0.5% tetracaine hydrochloride drops and a 4% lidocaine pledget (*n* = 31), 0.5% tetracaine hydrochloride drops alone (*n* = 31), or 4% cocaine (+ epinephrine 1/100000) drops alone (*n* = 31).

Rifkin et al. [14] included 60 patients randomized into three groups receiving one of three commonly used anesthetic methods: tetracaine HCl 0.5% gel, proparacaine HCl, or tetracaine HCl ophthalmic solution. A single drop of anesthetic was administered three times over a 5-minute period before intravitreal injection.

Kozak et al. [16] included 18 patients. The first group (*n* = 12) received intravitreal injections after both subconjunctival injection of 2% lidocaine and topical 2% lidocaine gel on separate occasions. The second group (*n* = 16) received either subconjunctival anesthesia (*n* = 8) or topical gel anesthesia (*n* = 8) before intravitreal injection of triamcinolone.

Shiroma et al. [17] included 260 patients prepared using a standardized injection protocol. Patients received proparacaine 0.5% 30 min before the procedure, followed by tropicamide 1% and 5% povidone-iodine. Topical anesthesia with lidocaine gel was applied 10 and 5 min before injection. Patients were randomized to receive lidocaine gel concentrations of 2%, 3.5%, 5%, 8%, or 12%. 

Reynolds et al. [18] included 36 patients undergoing intravitreal injections. Patients were randomized to treatment with additional topical proparacaine 0.5% versus control. All patients underwent conjunctival culture after one drop of topical proparacaine 0.5% was placed prior to injection. Half of the patients then received an additional drop of proparacaine followed by a second conjunctival culture, while the other half received povidone-iodine before the second culture. Intravitreal injection followed conjunctival cultures.

Wingelaar et al. [19] included 46 patients randomized into three groups. All groups received topical proparacaine drops and lidocaine gel as part of the standard anesthesia protocol. Group A (*n* = 16) received bromfenac 0.09% before injection and propylene glycol 0.6% after injection. Group B (*n* = 15) received propylene glycol 0.6% before injection and bromfenac 0.09% after injection. Group C (control group, *n* = 15) received propylene glycol 0.6% both before and after injection. 

Blaha et al. [20] included 24 patients, each receiving four intravitreal injections using four types of anesthesia: topical 0.5% proparacaine, topical 0.5% tetracaine, a pledget of 4% lidocaine, and a subconjunctival injection of 2% lidocaine. All patients received a drop of antibiotic (ofloxacin; Falcon, Fort Worth, TX) before and after the intravitreal injection. 

Lo et al. [21] included 60 patients undergoing repeated intravitreal injections. All patients first received one drop of topical proparacaine 0.5% followed by 5% povidone-iodine preparation. Patients were randomized to receive either a pledget soaked in proparacaine 0.5%, or a saline-soaked placebo pledget applied to the intended injection site for one minute before intravitreal injection. At the subsequent visit, each patient crossed over to the alternate intervention, allowing every participant to serve as his or her own control.

Yahalomi et al. [22] enrolled 239 consecutive patients undergoing intravitreal injections randomized into five distinct anesthesia protocol groups: lidocaine gel 3% (*n* = 40), lidocaine gel 10% (*n* = 47), topical oxybuprocaine 0.4% (*n* = 66), tetracaine HCl 1% drops (*n* = 35), or a combination of oxybuprocaine 0.4% and an ice patch applied for two minutes (*n* = 51).

### Patient-reported complications

In this section, we present the findings regarding patient-reported complications associated with the treatment, excluding patients who withdrew from the studies (*n* = 14). Complications were observed across several studies, often occurring immediately following the anesthetic application or during the subsequent intravitreal injection.

#### Anesthesia-related complications

Complications specifically linked to the anesthetic method were primarily reported following subconjunctival (SC) injections. In one study, 54% of eyes receiving SC lidocaine developed a subconjunctival hemorrhage (SCH) from the anesthesia itself [20]. Following the SC anesthetic injection, SCH extending more than two hours occurred in 58% of Group A (crossover group receiving both 2% lidocaine SC injection and 2% lidocaine topical gel) and 50% of Group B (single treatment group). Furthermore, conjunctival chemosis was reported in 83% of Group A and 75% of Group B following the SC injection [16].

#### Post-IVI injection complications

Additional complications were observed following the IVI procedure itself. One study reported that SCH was present in 70 eyes immediately after the IVI and in 82 eyes 30 min later. While SCH was less frequent in eyes treated with tetracaine and naphazoline drops, the difference was not statistically significant. Notably, SCH immediately after the IVI was more frequent in eyes treated with ranibizumab compared to bevacizumab, though this difference dissipated 30 min post-injection²³.

Regarding localized sensations, one study reported 14 cases of foreign body sensation following the use of topical lidocaine gel at varying concentrations. This sensation was least common in the 5% concentration group and most frequent in the 12% group, though no significant difference was found between the concentrations. The same study noted 11 total cases of SCH across the different gel concentrations (2% to 12%), again with no significant difference between the groups [17].

#### Studies reporting minimal complications

In contrast, several studies reported no significant treatment-related complications. Lo et al.²⁷ found no significant adverse events associated with either proparacaine-soaked pledgets or saline placebo pledgets during repeated IVIs. Yahalomi et al. [22] reported that no statistically significant differences were found between treatment groups regarding subconjunctival hemorrhage area (*p* = 0.35) or circumference (*p* = 0.30).

### Meta-analysis

#### Comparative efficacy of different anesthetic strategies for intravitreal injections (IVI)

The efficacy of the different anesthetic agents and their modes of administration was evaluated using the analog (Visual or Oral) scales ranging from 0 to 10 or 0 to 100 for pain. Pain scores were extracted as continuous variables (mean ± SD) and pooled as raw means (MRAW) on the 0–10 VAS. Analyses were performed using the *meta* package for proportional (single arm) pooling and the *metafor* package for prediction intervals where appropriate, in R (version 4.6). Because the included studies were predominantly single-arm and employed heterogeneous concentrations, formulations, and timing protocols, random-effects models were applied throughout, and between-study heterogeneity was quantified using I² and τ².

#### Topical lidocaine-based formulations

**Fig. 2:**
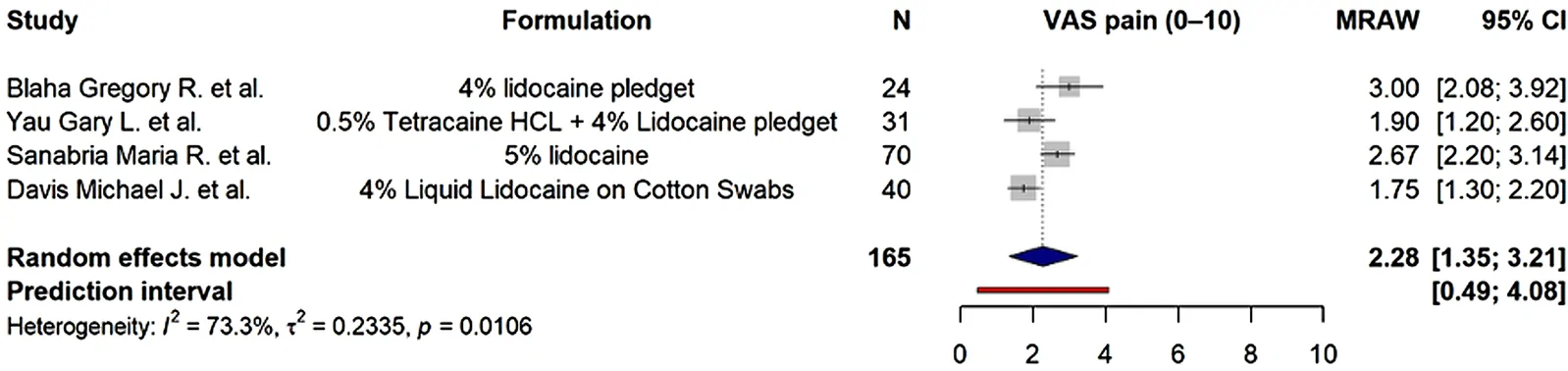
Pooled VAS pain scores for topical lidocaine formulations

Four studies provided usable data on topical lidocaine preparations, that includes drops, cotton-swab application, and pledgets (*N* = 165). The pooled mean VAS pain score was 2.28 (95% CI: 1.35–3.21), indicating only mild residual discomfort during intravitreal injection. The lowest scores came from 4% liquid lidocaine on cotton swabs (Davis et al., 1.75) and with the tetracaine-lidocaine pledget (Yau et al., 1.90). The 4% lidocaine pledget alone produced the highest score (Blaha et al., 3.00). The heterogeneity was moderate-to-high (I² = 73.3%, τ² = 0.23, *p* = 0.0106), which reflects differences in concentrations, contact times, and carrier formulation across studies. The prediction interval (0.49–4.08) remained within the lower half of the scale, suggesting that topical lidocaine can be expected to provide reliably mild pain scores in future settings. Overall, topical lidocaine preparations offer effective analgesia for IVIs (Fig. 2).

#### Gel-based formulations of lidocaine

**Fig. 3:**
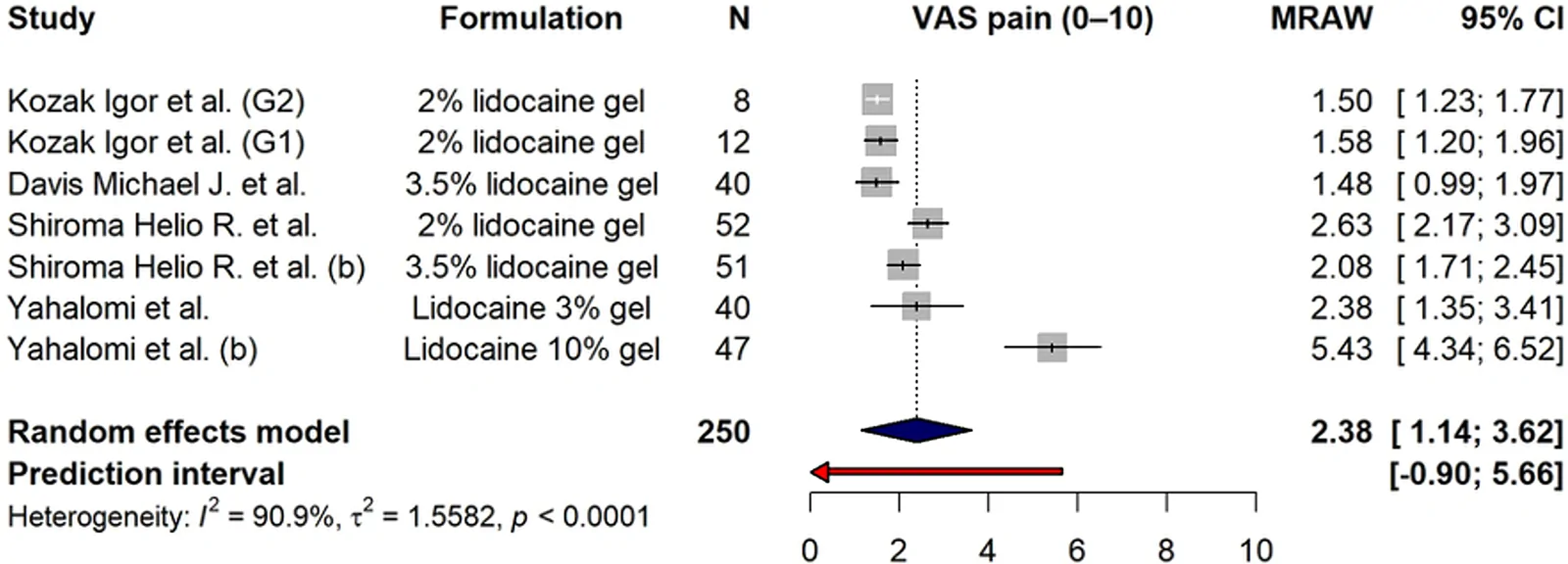
Pooled VAS pain scores for gel-based lidocaine formulations

Seven treatment arms reported gel-based lidocaine formulations with concentrations from 2% to 10% (*N* = 250). The pooled mean VAS score was 2.38 (95% CI: 1.14–3.62), again within the mild range and quite similar to the topical solutions. Most arms had scores between 1.48 and 2.63; the exception was the 10% lidocaine gel (Yahalomi et al., 5.43), which produced a higher pain and showed as a statistical outlier. This single high-concentration arm largely explains the very high heterogeneity observed (I² = 90.9%, τ² =1.5582, *p* < 0.0001) and the wide prediction interval (− 0.90 to 5.66). The negative lower bound is an artifact of the normal approximation rather than a meaningful value on the bounded VAS. Six of seven arms clustered between approximately 1.5–2.6 VAS, while the 10% lidocaine gel arm produced substantially higher pain scores and was the principal contributor to heterogeneity (Fig. 3).

#### Comparison between different lidocaine formulations

**Fig. 4:**
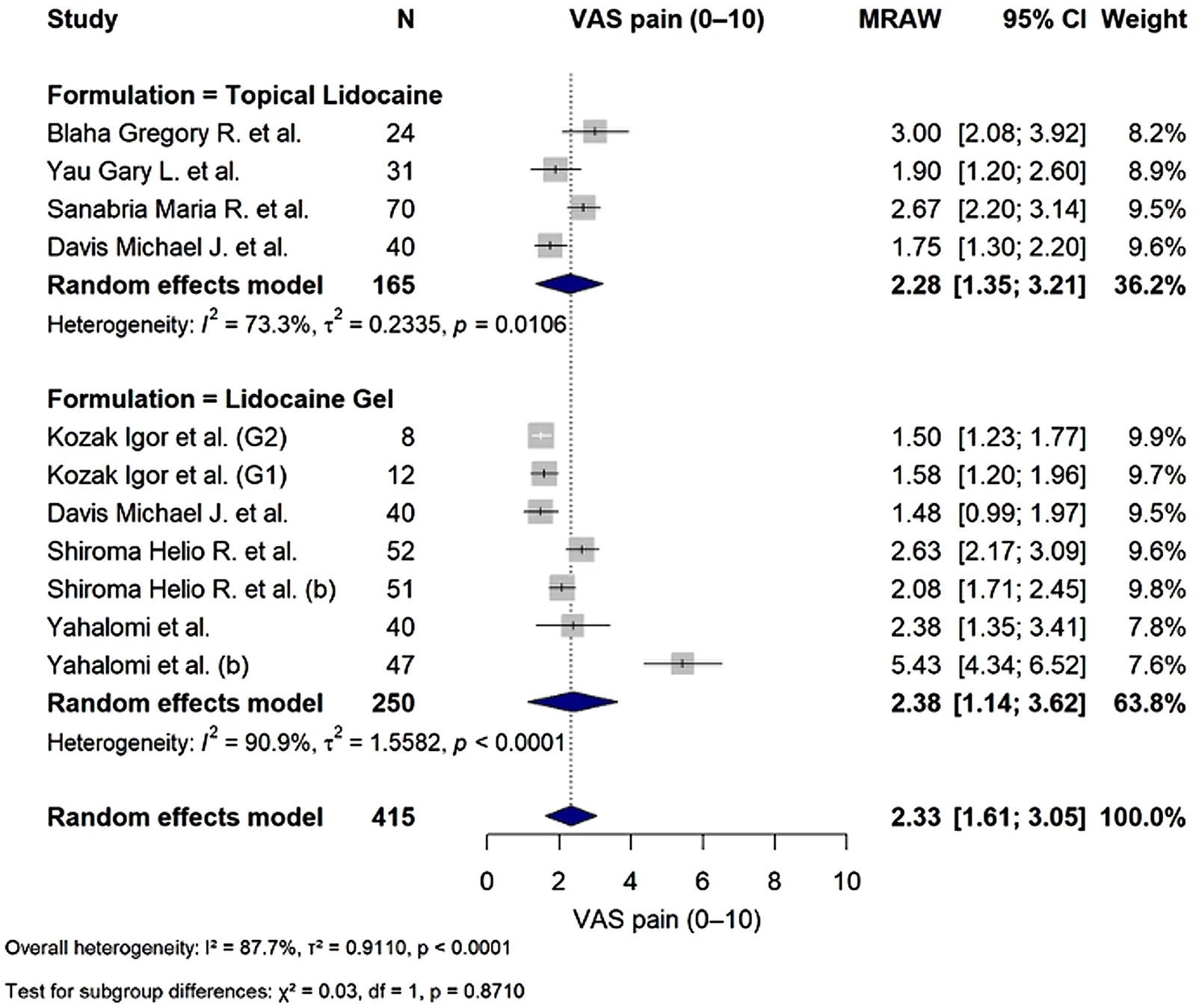
Subgroup comparison of topical versus gel-based lidocaine

We then compared topical and gel-based lidocaine within a single subgroup model. The pooled estimates were similar 2.28 for topical Lidocaine (95% CI: 1.35–3.21) and 2.38 gel-based lidocaine (95% CI: 1.14–3.62), with an overall pooled mean of 2.33 (95% CI: 1.61–3.05). The subgroup test showed no statistically significant difference between the two formulations (χ² = 0.03, df = 1, *p* = 0.8710). These findings suggest that topical and gel-based lidocaine are comparable analgesia for IVIs, so the choice between them may depend on clinician preference, cost, availability, and ease of application (Fig. 4).

#### Tetracaine

**Fig. 5:**
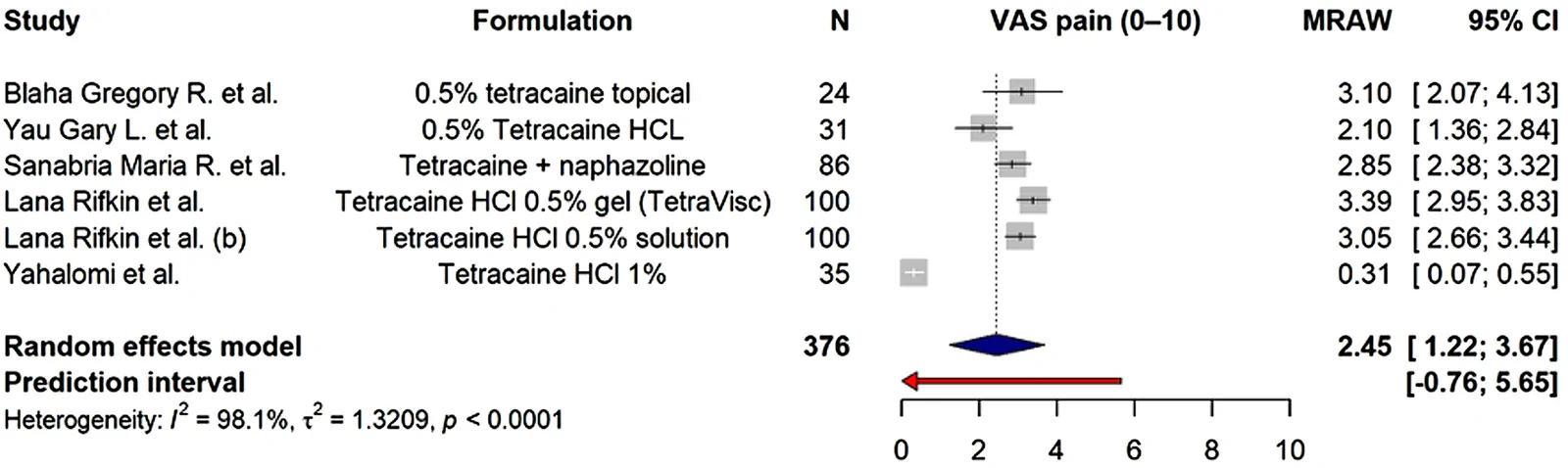
Pooled VAS pain scores for tetracaine formulations

Six arms evaluated tetracaine, either alone or in combination with another agent (*N* = 376). The pooled mean VAS score was 2.45 (95% CI: 1.22–3.67), comparable to the lidocaine estimates. Most formulations including the topical solution, the 0.5% gel (TetraVisc), and tetracaine with naphazoline were clustered between 2.10 and 3.39, while the 1% tetracaine arm (Yahalomi et al., 0.31) showed substantially lower pain scores than the other studies and contributed to between-study heterogeneity. The combination of 0.5% tetracaine HCl with 4% lidocaine did not appear to provide additional benefit over tetracaine monotherapy. Similarly, topical and gel-based tetracaine showed broadly comparable analgesic effects. Heterogeneity was high (I² = 98.1%, τ² = 1.3209, *p* < 0.0001) and the prediction interval was wide (-0.76 to 5.65). As the included studies did not stratify by indication for IVI, part of this dispersion likely reflects differences in case-mix and protocol rather than the agent itself (Fig. 5).

#### Topical proparacaine

**Fig. 6:**
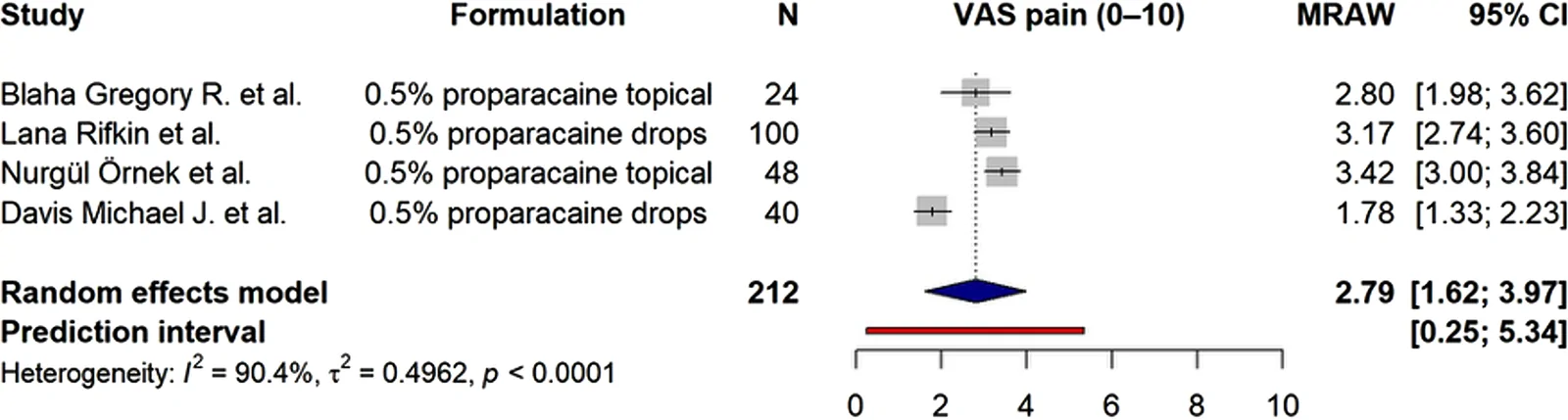
Pooled VAS pain scores for topical proparacaine

Four studies reported topical proparacaine, either as eye drops or by topical application (*N* = 212). The pooled mean VAS score was 2.79 (95% CI: 1.62–3.97), the highest among the three agents though still within the mild range. Three studies showed similar mean scores from 2.80 to 3.42, while Davis et al. reported a lower score 1.78; all four estimates lay in a direction consistent with effective surface anesthesia. Heterogeneity was high (I² = 90.4%, τ² = 0.49, *p* < 0.0001), and the prediction interval ranged from 0.25 to 5.34. Overall, Proparacaine appears to provide an adequate analgesia for IVIs, although it has higher pooled pain scores than lidocaine or tetracaine (Fig. 6).

#### The comparative efficacy of lidocaine versus proparacaine versus tetracaine

**Fig. 7:**
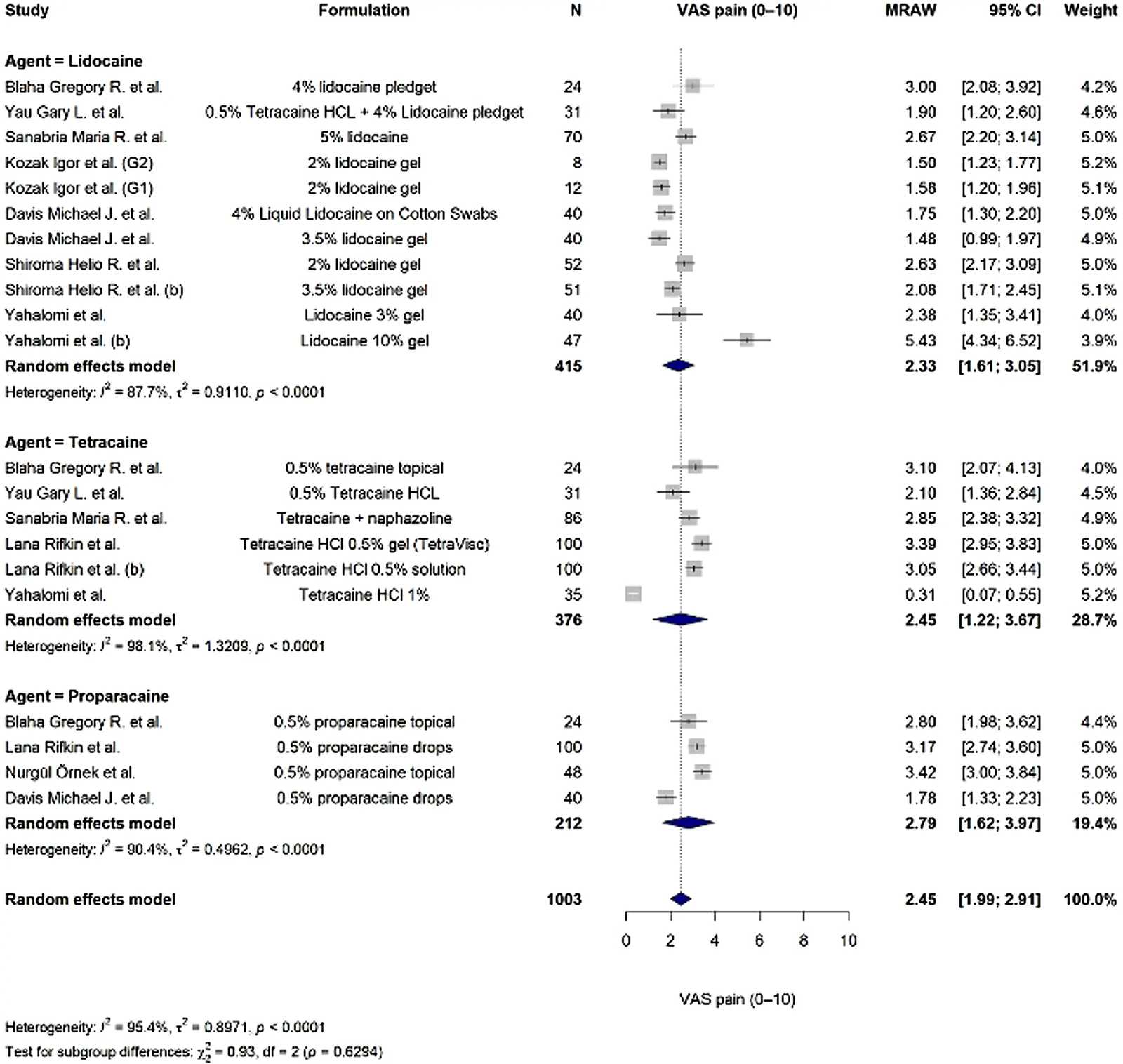
Subgroup comparison of lidocaine, tetracaine, and proparacaine

The three agents were compared within a single subgroup model (*N* = 1003). Lidocaine had the lowest pooled mean VAS scores at 2.33 (95% CI: 1.61–3.05), followed by tetracaine at 2.45 (95% CI: 1.22–3.67) and proparacaine at 2.79 (95% CI: 1.62–3.97). The overall pooled estimate across all of the three was 2.45 (95% CI: 1.99–2.91). Translated into analgesic efficacy, this corresponds to a trend of lidocaine > tetracaine > proparacaine. However, the confidence intervals overlapped considerably, and the subgroup differences were statistically non-significant (χ² = 0.93, df = 2, *p* = 0.62). In addition, the differences between them were small, with less than 0.5 points separating the pooled VAS scores. and unlikely to represent a clinically meaningful advantage for any single agent (Fig. 7).

#### Other anesthetic agents

**Fig. 8:**
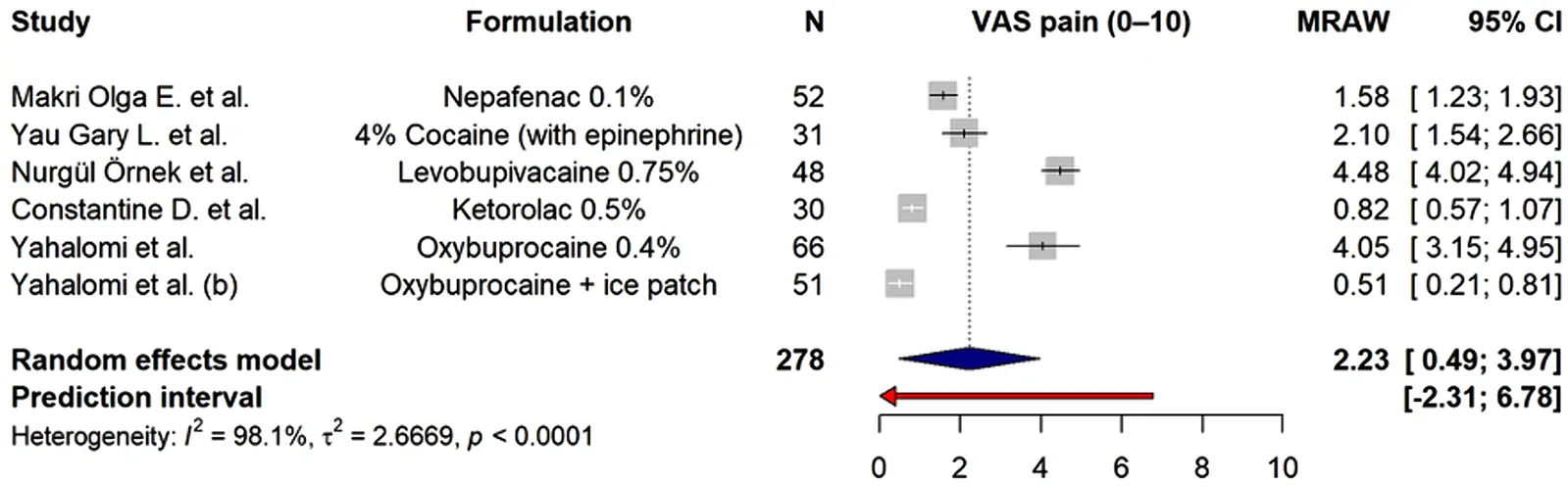
Pooled VAS pain scores for other anesthetic agents

Several studies investigated alternative agents, including NSAIDs (nepafenac, ketorolac), a long-acting amide anesthetic (levobupivacaine), cocaine with epinephrine, and oxybuprocaine with or without an ice patch (*N* = 278). The lowest pain scores were achieved with oxybuprocaine combined with an ice patch (Yahalomi et al., 0.51) and with 0.5% ketorolac (Constantine et al., 0.82); notably, adding an ice patch lowered the oxybuprocaine score from 4.05 to 0.51, underscoring the value of simple adjunctive cooling. At the other extreme, levobupivacaine (Nurgül et al., 4.48) and oxybuprocaine alone (4.05) were the least effective, while nepafenac (1.58) and cocaine with epinephrine (2.10) produced intermediate effects. Given this dispersion, heterogeneity was extreme (I² = 98.1%, τ² = 2.6, *p* < 0.0001), and the pooled estimate of 2.23 (95% CI: 0.49–3.97), with a prediction interval spanning (-2.31 to 6.78), is best regarded as inconclusive. These agents may nonetheless have a role as adjuncts, particularly when paired with physical cooling (Fig. 8).

#### Comparative safety (Incidence of subconjunctival hemorrhage)

**Fig. 9:**
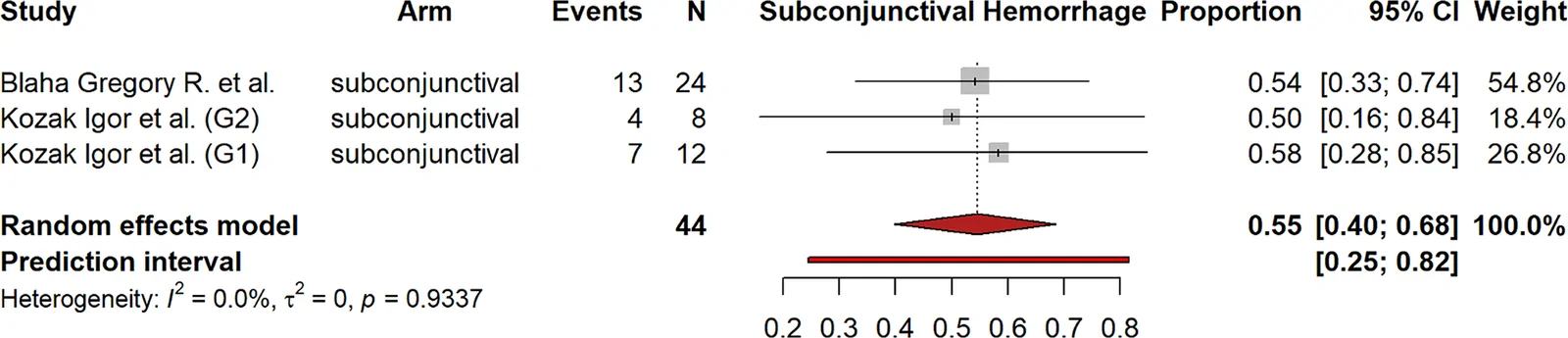
Pooled incidence of subconjunctival hemorrhage

Safety was assessed by pooling the incidence of subconjunctival hemorrhage across three cohorts involving 44 patients who received anesthetic through the subconjunctival route. The pooled proportion was 0.55 (95% CI: 0.40–0.68), indicating that slightly more than half of the patients developed a subconjunctival hemorrhage. heterogeneity was absent (I² = 0.0%, τ² = 0, *p* = 0.933), and the prediction interval (0.25–0.82) was relatively narrow, indicating a consistent event rate across studies. However, subconjunctival hemorrhage following IVI is typically minor and self-limiting (Fig. 9).

#### Sensitivity analyses

Leave-one-out and sensitivity analyses were conducted to assess whether any specific study had a disproportionate effect on the pooled estimates.

**Fig. 10:**
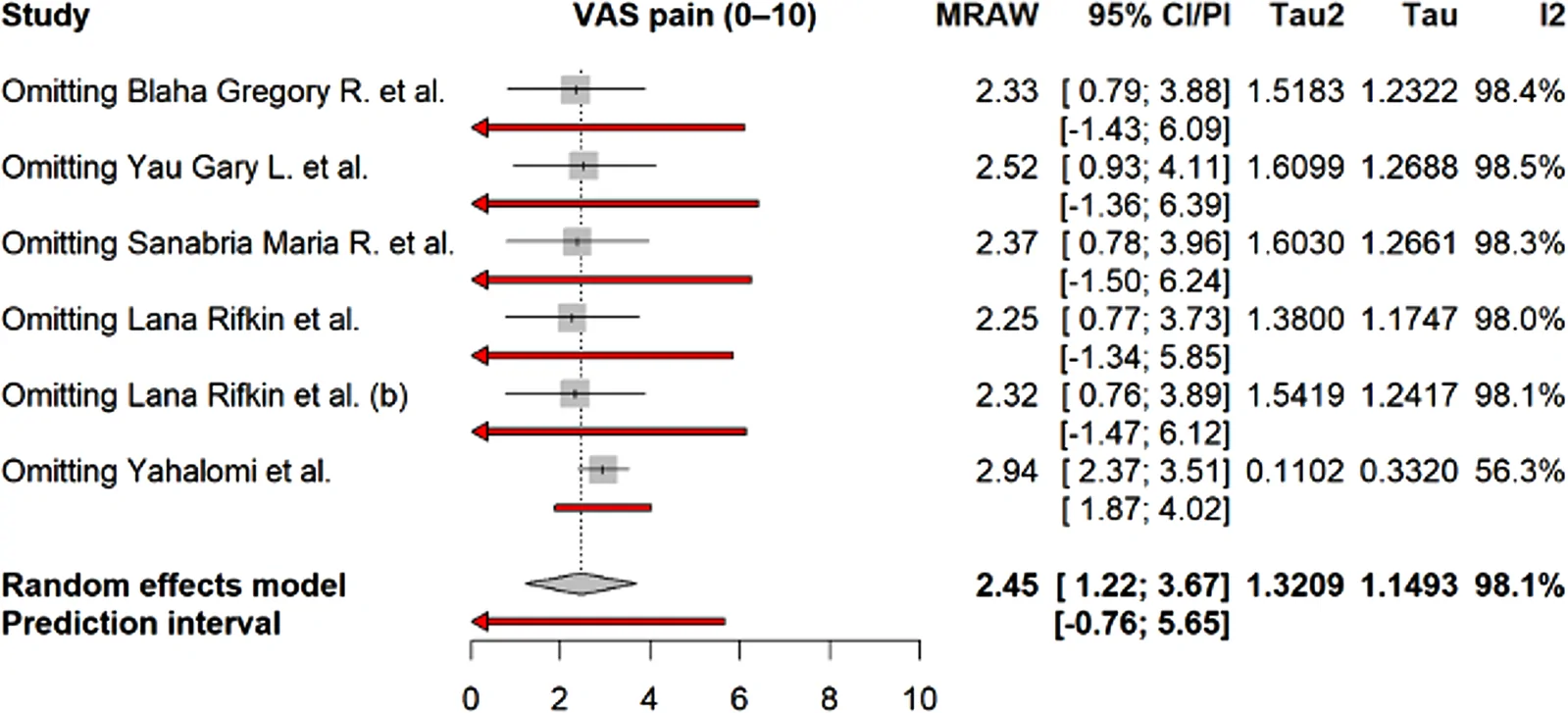
Leave-one-out sensitivity analysis for tetracaine formulations

**Fig. 11:**
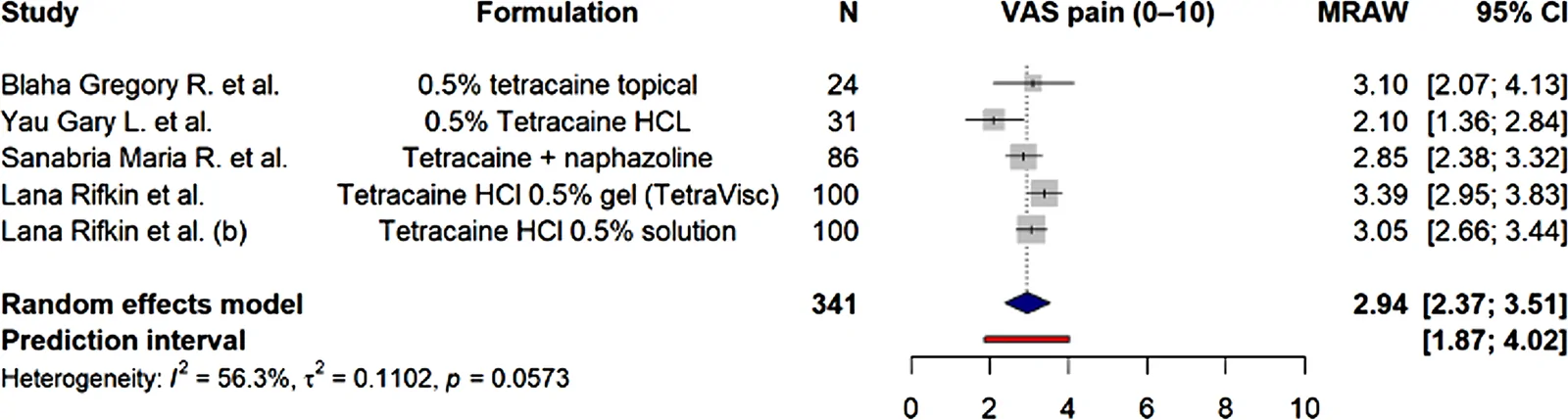
Sensitivity analysis excluding the 1% tetracaine arm

For tetracaine formulations, the leave-one-out analysis identified the 1% tetracaine arm reported by Yahalomi et al. as the main contributor to heterogeneity. Omission of this arm reduced heterogeneity from I² = 98.1% to 56.3%, while exclusion of the other studies produced only minor changes (Fig. 10). A further sensitivity analysis was therefore performed after excluding this arm (Fig. 11). The pooled VAS score increased from 2.45 (95% CI: 1.22–3.67) to 2.94 (95% CI: 2.37–3.51), while τ² decreased from 1.32 to 0.11. The prediction interval also narrowed from − 0.76 to 5.65 in the primary analysis to 1.87 to 4.02 after exclusion, indicating greater consistency across the remaining studies.

**Fig. 12:**
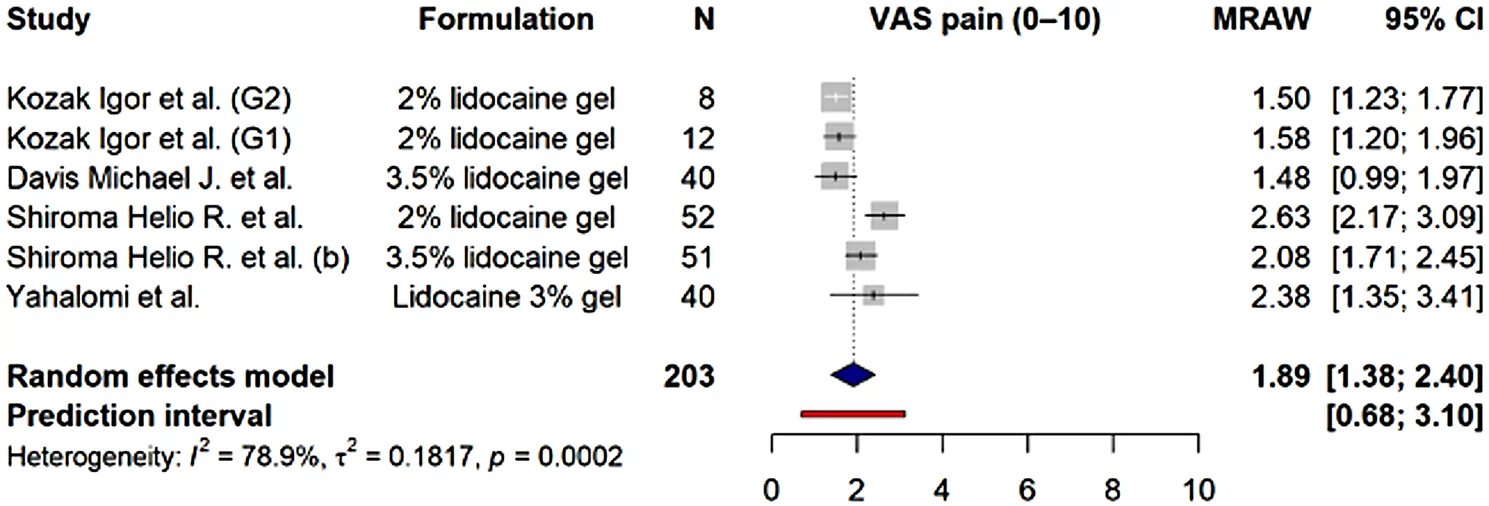
Sensitivity analysis excluding the 10% lidocaine gel arm

For lidocaine gel formulations, excluding 10% lidocaine gel arm reduced heterogeneity from I² = 90.9% to 78.9% and lowered the pooled VAS score from 2.38 (95% CI: 1.14–3.62) to 1.89 (95% CI: 1.38–2.40) (Fig. 12). This suggests that the 10% lidocaine gel arm contributed meaningfully to between-study heterogeneity, although its removal did not change the overall interpretation that lidocaine gel provides effective analgesia for IVIs.

## Discussion

This systematic review and meta-analysis synthesized data from 14 randomized controlled trials to evaluate the efficacy and safety of various topical anesthetic methods for intravitreal injections. Our findings indicate that while multiple topical anesthetic agents effectively manage procedural pain, no single agent demonstrated clear superiority, with lidocaine, tetracaine, and proparacaine all providing comparable analgesia.

The analysis of topical lidocaine-based formulations (including drops, pledgets, and gels) demonstrated consistent effectiveness, with pooled mean Visual Analog Scale (VAS) scores indicating only mild residual pain [15, 17]. Similarly, tetracaine formulations showed broadly comparable efficacy to lidocaine [12, 15]. Proparacaine, while effective, exhibited the highest pooled mean VAS scores among the three primary agents, though these remain within a clinically acceptable range for routine practice [11, 18]. Furthermore, direct comparison between topical and gel-based lidocaine formulations revealed no significant difference (*p* = 0.87), suggesting the choice between them may be guided by availability and ease of application rather than analgesic efficacy [23]. Notably, the 10% lidocaine gel arm was identified as a statistical outlier (VAS 5.43), and was substantially higher than all other arms, which clustered between 1.5 and 2.6 [17]. Sensitivity analysis excluding this arm reduced the pooled VAS from 2.38 to 1.89, suggesting that higher concentrations do not confer analgesic benefit and may increase discomfort.

A notable finding in our review, as highlighted in studies such as Sanabria et al. (2013) [10], is the comparative performance of different pre- and post-injection protocols. Sanabria et al. evaluated two preoperative regimes: 0.5% tetracaine combined with 0.05% naphazoline versus 5% lidocaine. They observed no statistically significant difference in pain scores between these regimes immediately post-IVI (*p* = 0.73) or at 24 h (*p* = 0.979). Furthermore, their comparison of post-injection protocols (tobramycin alone versus tobramycin combined with 0.1% diclofenac) showed no significant difference in pain reduction (*p* = 0.46), suggesting that simple antibiotic prophylaxis may be sufficient without added topical NSAIDs for routine post-operative comfort.

When considering adjunctive therapies, our review identified significant benefits from physical interventions. For instance, the combination of oxybuprocaine with an ice patch, as reported by Yahalomi et al., yielded some of the lowest pain scores in our dataset, highlighting the potential value of simple, non-pharmacological cooling adjuncts [22, 24]. In contrast, levobupivacaine produced the highest pain scores among all agents evaluated (VAS 4.48), suggesting it offers no advantage over standard topical agents and should not be considered a preferred alternative for routine IVI anesthesia [11]. 

Regarding subconjunctival anesthesia, our review supports a cautious approach. While studies like Kozak et al. (2005) [16] allow for direct comparison between subconjunctival injections and topical gel formulations, our safety analysis confirms that subconjunctival lidocaine is associated with a high incidence of subconjunctival hemorrhage (pooled proportion 0.55) and chemosis [20]. Given that topical methods provide effective analgesia with significantly fewer complications, topical strategies should remain the preferred approach for routine clinical practice.

Our meta-analysis suggests that clinicians may prioritize anesthetic methods based on availability, patient comfort, and institutional protocols rather than expecting one topical agent to be significantly more effective than others. Future research should focus on refining standardized, low-risk protocols that integrate simple, effective adjuncts like cooling to further optimize the patient experience.

## Conclusion

In conclusion, this systematic review and meta-analysis demonstrates that while various topical anesthetic agents are effective in managing procedural pain during intravitreal injections, no single topical agent shows clear clinical superiority over the others. All primary agents consistently achieve mild mean pain scores, and the choice of agent may largely depend on clinical availability, cost, and patient preference. Furthermore, our findings indicate that subconjunctival anesthesia is associated with a higher risk of adverse events, such as subconjunctival hemorrhage and chemosis, compared to topical approaches. Consequently, topical anesthetic strategies should remain the preferred method for routine intravitreal injections. Simple, non-pharmacological adjuncts, particularly physical cooling, offer a safe and effective means of further enhancing patient comfort. Future research efforts should prioritize the standardization of low-risk anesthetic protocols to optimize the overall patient experience in long-term intravitreal therapy.

## Data Availability

No datasets were generated or analysed during the current study.

## Declarations

### Presentation(s) or awards at a meeting

None to declare.
